# Aligning evidence for the genesis of visual gamma oscillations

**DOI:** 10.1371/journal.pbio.3001701

**Published:** 2022-06-28

**Authors:** Brett L. Foster, Eleonora Bartoli

**Affiliations:** 1 Department of Neurosurgery, Perelman School of Medicine, University of Pennsylvania, Philadelphia, Pennsylvania, United States of America; 2 Department of Neurosurgery, Baylor College of Medicine, Houston, Texas, United States of America

## Abstract

This Primer explores the implications of a recent PLOS Biology study showing that gamma synchrony in visual cortex is disrupted when small discontinuities are added to visual stimuli. This suggests that gamma synchrony is highly sensitive to regularities in the visual world and potentially play a role in predictive processing.

## Teaser

New findings in *PLOS Biology* show that visual gamma oscillations are greatly attenuated by small spatial discontinuities in visual stimuli, suggesting that their genesis occurs in response to predictable regularities in the visual world.

Periodic fluctuations in the brain’s electrical activity, known as oscillations, have been the focus of neuroscientists for almost a century. These oscillations occur at different frequencies across brain regions and in response to a variety of stimuli or behaviors [1]. Of particular research focus has been the striking high-frequency (30 to 80 cycles/second) gamma oscillations that occur within visual cortex. While a highly robust phenomenon across species, debate continues as to the functional significance of these visual gamma oscillations. In this issue of *PLOS Biology*, Shirhatti and colleagues [2] show that even subtle tweaks in the structure of specific gamma inducing stimuli greatly reduces the amplitude of this oscillation. These findings present important challenges for theories of gamma’s functional role in visual cortex. However, as the authors show, these findings intimately relate to key physiological properties of neural circuits within visual cortex, opening new avenues for understanding gamma’s genesis and function.

**Fig 1:**
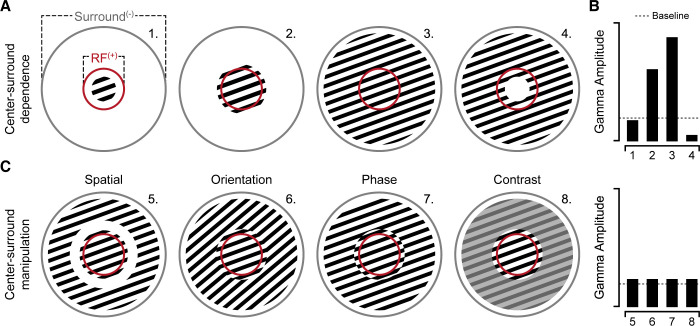
RF center-surround factors that influence gamma amplitude. (**A**) Simplified schematic depiction of RF center (+ excitatory) and surround (− inhibitory), with related changes in gamma amplitude shown in (**B**; upper). High contrast gratings smaller than the RF center do not induce gamma oscillations beyond baseline levels (1). Grating stimuli must extend through the RF center to increase gamma amplitude (2), which is maximally driven when coherently falling on both RF and surround (3). Gratings that predominately fall on the surround and not the RF center will reduce gamma amplitude below baseline levels (4; likely due to a predominance of inhibition without RF excitatory drive) [6]. (**C**) Grating center-surround manipulations performed by Shirhatti and colleagues (2). Even if grating stimuli fall upon both RF center and surround, small spatial (5), orientation (6), phase (7) or contrast (8) discontinuities between the center-surround greatly attenuate gamma amplitude (B; lower) from levels observed for contiguous gratings of the same size (3). RF, receptive field.

Primary visual cortex (V1) is an essential structure of the visual system, containing neurons that increase their spiking activity in response to basic visual features such as lines and colors. When these features are presented as uniform patterns (e.g., striped lines or gratings; see Fig 1), groups of neurons fire repeatedly together. This synchrony of neural firing, along with related physiological events, appears as large amplitude gamma frequency oscillations in the electrical field potential. It has been proposed that gamma may serve to temporally align neural activities, either within a visual area to support the perceptual binding of line features in visual space or across visual areas to support neural communication along processing pathways [3]. Experimental and computational work has grown in examining these and other claims regarding the origins and significance of gamma oscillations in vision and cognition.

As the grating stimuli that induce strong gamma oscillations reflect a common stimulus in visual neuroscience, many studies have examined how changes in the attributes of gratings impact neural responses in visual cortex, including gamma. It is now clear that changing the properties of grating stimuli can parametrically impact the amplitude and/or frequency of induced visual gamma [4], consistently across species. Together, accumulating evidence suggests visual gamma is particularly sensitive to the uniformity of grating stimulus properties. Most critical is perhaps the observation that the use of more complex and irregular visual stimuli, such as photographs (i.e., images of natural phenomena), do not consistently induce gamma oscillations. This important progress in elucidating the stimulus conditions that optimally drive gamma raises 2 challenges: (i) Is there a common principle that underlies stimuli which induce visual gamma? (ii) What is the functional role of gamma in light of this principle? Vinck and Bosman [5] proposed that gamma oscillations occur under conditions where visual input features to the receptive field (RF) align with the local surround (Fig 1A). More specifically, gamma is enhanced when RF input is highly predicted based on the surround. This theory accounts for many prior observations related to visual gamma and integrates theories of predictive coding with known RF center-surround neurophysiology [5].

In their work, Shirhatti and colleagues [2] directly tested this theory by quantifying changes in visual gamma during systematic manipulation of grating center-surround continuity, in spatial, orientation, phase, and contrast domains (see Fig 1C). To do so, they first carefully mapped RFs within V1 of awake macaque monkeys via high-density intracortical microelectrode recording arrays. Next, they presented visual grating stimuli centered on the RFs and subsequently manipulated parts of the grating falling at or beyond the center-surround transition zone—directly testing how small discontinuities between the RF and its surround impacted gamma. The authors first tested the simplest form of discontinuity by adding a progressively larger ring (annulus) between the RF center and surround. Introduction of this discontinuity greatly reduced the amplitude of gamma oscillations, compared to the control uniform grating stimulus. This particular manipulation is expected based on prior observations [6], but the authors go on to examine other more subtle discontinuities by progressively misaligning grating features (see Fig 1). Specifically, the authors show that visual gamma amplitude is parametrically reduced as the degree of orientation, phase, or contrast offset between the RF center and its surround is increased. In contrast to changes in gamma, average population spiking activity showed more modest changes, although importantly in the opposing direction. This is consistent with the well-known suppression of population spiking activity for coherent center-surround stimuli. Together, these data suggest that stimuli which maximally drive gamma oscillations are typically associated with lowered, and more temporally synchronous, spiking patterns.

This consistent set of findings from Shirhatti and colleagues [2] provides critical support for the center-surround prediction theory of gamma genesis [5], which was also recently tested for uniform color stimuli [7] and complex images [8]. These findings prompt us to reconsider prior theories, such that visual gamma’s primary function may not be that of a general mechanism for coordinating visual circuit interactions, but rather, a contextually specific mechanism for the efficient processing of redundant (i.e., predictable) feature configurations in the visual world [5]. Together, there is now a growing consensus for the types of stimulus configurations optimal for gamma genesis in visual cortex, allowing for impressive prediction of observed gamma amplitude based on knowledge of the stimulus input and RF [8,9]. A critical next step will be examining the occurrence of such stimulus configurations within natural scene statistics and the resulting implications for the functional role of gamma oscillations in natural vision. As the authors note, stimulus-based models are, in part, agnostic to physiological mechanisms. What then are the specific circuit dynamics which underlie gamma genesis? Building on a large literature, Shirhatti and colleagues [2] show that a well-known model of coupled excitatory and inhibitory neurons is able to recapitulate many of their experimental results. However, such models lack specific incorporation of visual circuit connectivity and related center-surround computations, which are essential to generating new predictions of gamma genesis under more complex multifeature conditions [10] of natural vision. Development of more biophysically realized models of visual cortex will be critical not only for understanding the physiological genesis of gamma, but also for the neurobiological significance of this intriguing phenomenon in visual circuits.
